# Interleukin-10/lymphocyte ratio predicts mortality in severe septic patients

**DOI:** 10.1371/journal.pone.0179050

**Published:** 2017-06-19

**Authors:** Xi Li, Zhiheng Xu, Xiaoqing Pang, Yongbo Huang, Baoxin Yang, Yuanyuan Yang, Kangxie Chen, Xiaoqing Liu, Pu Mao, Yimin Li

**Affiliations:** 1 State Key Laboratory of Respiratory Disease, Guangzhou Medical University, Guangzhou, Guangdong, China; 2 Intensive Care Unit, The People’s Hospital Of Leshan, Sichuan, China; 3 Intensive Care Unit, The First Affiliated Hospital of Guangzhou Medical University, Guangzhou, Guangdong, China; 4 Department of Infection Control, The First Affiliated Hospital of Guangzhou Medical University, Guangzhou, Guangdong, China; Azienda Ospedaliero Universitaria Careggi, ITALY

## Abstract

**Background:**

Immunosuppression is common even in the early stage of severe sepsis. Interleukin-10 (IL-10) secretion and lymphocyte exhaustion are the main features of sepsis-induced immunosuppression. However, the relationship between IL-10 and the lymphocyte is still unclear. We investigated if IL-10/lymphocyte ratio (IL10LCR) were associated with mortality in severe septic patients.

**Methods:**

Adult patients with severe sepsis admitted to ICU of the First Affiliated Hospital of Guangzhou Medical University were identified from October 2012 to August 2013. Within 24 hours of ICU admission, peripheral whole blood was collected for the measurement of IL-10 using commercial multiplex bead-based assay kits and determination of lymphocyte count from laboratory data. The primary outcome was 28-day mortality.

**Results:**

A total of 63 severe sepsis patients were identified. There were 20 (32%) patients died within 28 days. IL10LCR in non-survival patients was significantly higher than survival patients (median (IQR) 36.78 (12.34–79.63) ng/ml^2^ versus 11.01(5.41–27.50) ng/ml^2^, *P* = 0.002). Correlation analysis showed that IL10LCR was significantly correlated with APACHE II score (Spearman’s rho = 0.424, *P*<0.001). The receiver operating characteristic (ROC) curves showed the area under the curve was 0.749 for IL10LCR level to predict 28-day mortality with sensitivity and specificity at 70.0% and 74.4%, respectively. At an optimal cutoff of 23.39ng/ml^2^, Kaplan-Meier curve showed survival in patients with IL10LCR level above 23.39ng/ml^2^ was significantly lower than in patients with IL10LCR level less than 23.39ng/ml^2^ (*P* = 0.001 by log-rank test).

**Conclusion:**

IL10LCR level is significantly associated with the severity and outcome of severe septic patients. It may serve as a biomarker for sepsis-induced immunosuppression.

## Introduction

Sepsis is the one of the leading causes of mortality in intensive care units (ICUs). The mortality of sepsis is around 25%-30% and the current treatments of sepsis are very limited[1,2]. Early studies suggested that uncontrolled “cytokine storm” contribute to major mortality of severe sepsis. However, the anti-inflammatory therapies have failed, showing no improvement in clinical outcomes[3]. Nowadays, it is believed that sepsis initiates a complex immunologic response to upsetting the balance between pro-inflammatory and anti-inflammatory processes, leading to immunosuppressive phase and resulting in uncontrolled primary infection or secondary hospital-acquired infections, which contributes to mortality increasing[4].

The mainly features of sepsis-induced immunosuppression was overproduction of anti-inflammatory cytokines and apoptosis-related immune cells[5]. Interleukin-10 (IL-10) is one of the most important anti-inflammatory cytokines, whose concentrations in the blood rang from 12pg/ml to 2400pg/ml in septic patients[6]. Previous researches reported that higher plasma IL-10 concentration contributed to higher mortality of sepsis[7,8]. Additionally, lymphocyte exhaustion, including CD4 and CD8 T cells, B cells, is common in septic patients[9,10]. Drewry *et*.*al* showed that persistent lymphopenia on the fourth day after diagnosis of sepsis can predict mortality[11]. Therefore, higher IL-10 and lymphopenia are important in sepsis-induced immunosuppression.

However, the relationship between IL-10/lymphocyte ratio(IL10LCR) and the mortality of severe sepsis patients remains unknown. Here, we investigated whether IL10LCR is associated with the outcome of severe sepsis.

## Methods

### Study design

We conducted a single-center, retrospective cohort study. Patients were recruited from the intensive care unit (ICU) of the First Affiliated Hospital of Guangzhou Medical University from October 2012 to August 2013. Written informed consent was obtained from their relatives. The study was approved by the Ethic Review Committee of the First Affiliated Hospital of Guangzhou Medical University.

### Study setting and population

All the patients admitted to the hospital with severe sepsis were included. Sepsis was defined as the presence of documented or clinically diagnosed infection with at least two of the following criteria: a) core temperature above 38°C or below 36°C; b) heart rate of more than 90 beats per minute; c) respiratory rate more than 20 breaths per minute or partial pressure of carbon dioxide below 32 mmHg; and d) leukocytosis (white blood cell count >12×10^9^/L) or leukopenia (white blood cell count <4×10^9^/L) or more than 10% bands in peripheral blood. Severe sepsis was defined as septic patients with at least one organ dysfunction[12].

Exclusion criteria were as follows: a) age<18 year, b) hematological or immunological disease, c) treatment with radiation therapy or chemotherapy for an underlying malignancy within 6 months prior to hospital admission, d) continuous administration corticosteroids (at least 0.5 mg/kg per day of prednisolone or an equivalent drug) at least a month during the 3 months, d) no plasma sample was collected within 24 hours.

### Plasma interleukin-10 measurements

Plasma samples were collected from each patient at the time of enrollment. Whole blood was collected in sodium citrate tubes and centrifuge at 1,000 ×g for 10 minutes. Plasma was removed and frozen at -80°C until use.

The plasma level of Interleukin-10 was measured using commercial multiplex bead-based assay kits (Bio-Plex Cytokines Assay, Bio-Rad Inc., USA) according to the manufacturer’s instructions[13].

### Data collection

Lymphocyte counts were recorded from laboratory data for the first day admitted to ICU. We also collected the following data: age, sex, underlying disease or comorbidities, the source of infection, the white blood cell count, neutrophil count, Acute Physiology and Chronic Health Evaluation II (APACHE II) score, the length of mechanical ventilation, the length of ICU and hospital stays. The primary outcome for the study was the 28-day mortality in ICU. All the data was recorded at the time of plasma samples collection.

### Statistical analysis

Quantitative data were expressed as mean and standard deviation (SD) for normal distribution, while median and interquartile range (IQR) for abnormal distribution. Qualitative data were presented as numbers(%). For continuous variables, two-group comparisons were performed using unpaired t-tests or Mann-Whitney tests according to test of normality. Spearman correlation coefficient was used to test the correlations between IL10LCR levels and other clinical variables. Receiver operating characteristic (ROC) curve was used to determine the sensitivity and specificity of prediction of IL10LC for clinical outcomes. Kaplan-Meier curves were performed by using IL10LCR groups as strata and compared by log-rank test. To identify risk factors for 28-day mortality, a simple and multiple Cox proportional hazards regression model was performed. All analyses and figures were performed by using SPSS 18.0 (SPSS Inc., Chicago, IL) or Prism 5.0 (Graphpad Software, La Jolla, CA). A *P*<0.050 (two-sided) was considered statistically significant.

## Results

### Study population

**Fig 1:**
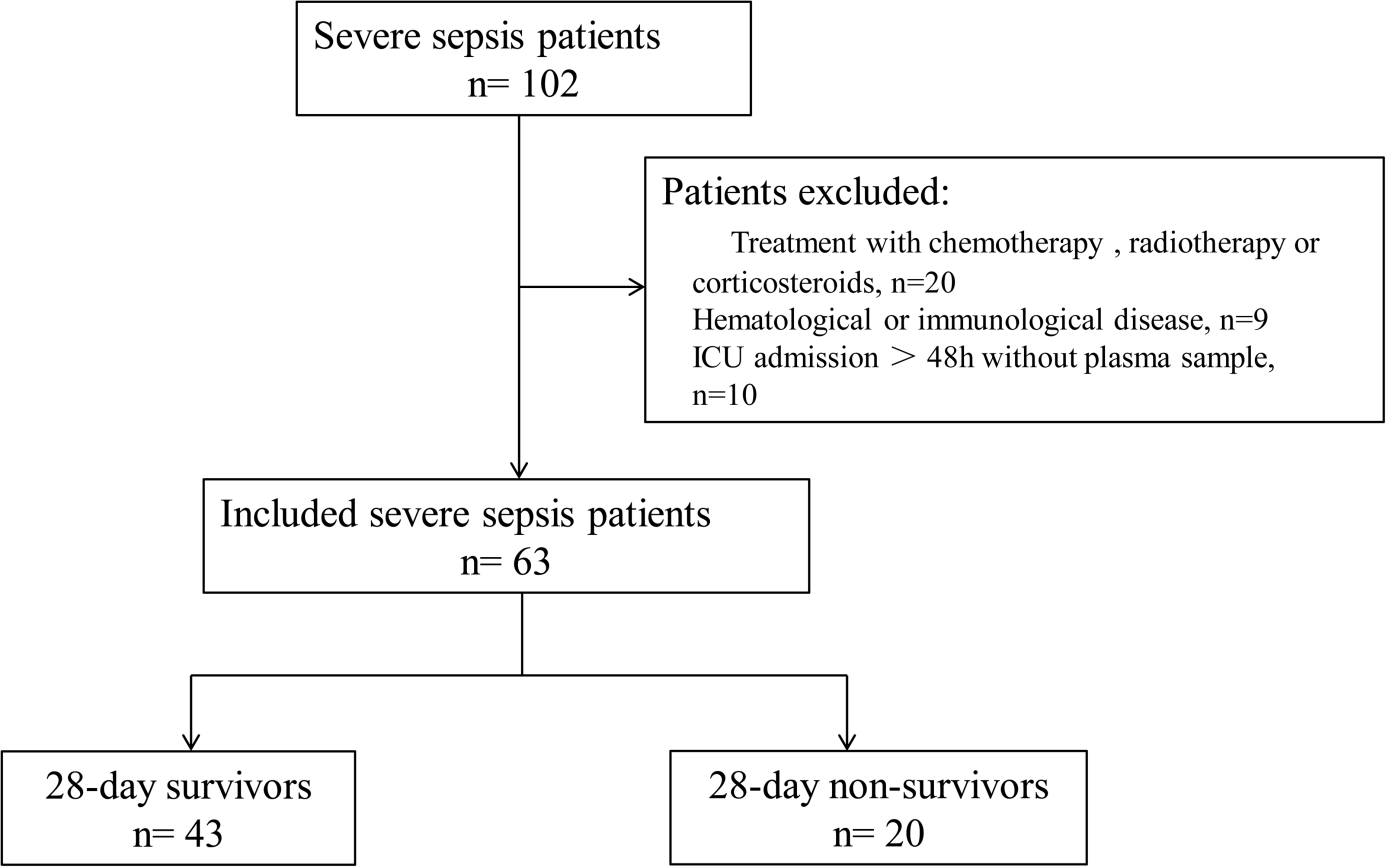
Flowchart of included and excluded patients.

There were 102 patients who met the criteria. However, 29 patients were excluded because of hematological or immunological disease, use of chemotherapy, radiation therapy or use of glucocorticoids. Ten patients were also excluded because of no plasma samples within 24 hours ICU admission. Finally, a total of 63 severe sepsis patients were included. Among them, 58(92%) patients had lung infection while 54(86%) patients had definitely presented of a microbiologically documented. There were 20(32%) patients died within 28-day follow-up (Fig 1).

### Comparison of clinical parameters and IL10LCR between the survivor and non-survivors

Patients were classified as survivors or non-survivors according to their 28-day clinical outcome of sepsis. No significant difference was observed between survivors or non-survivors in terms of age and sex.

**Fig 2:**
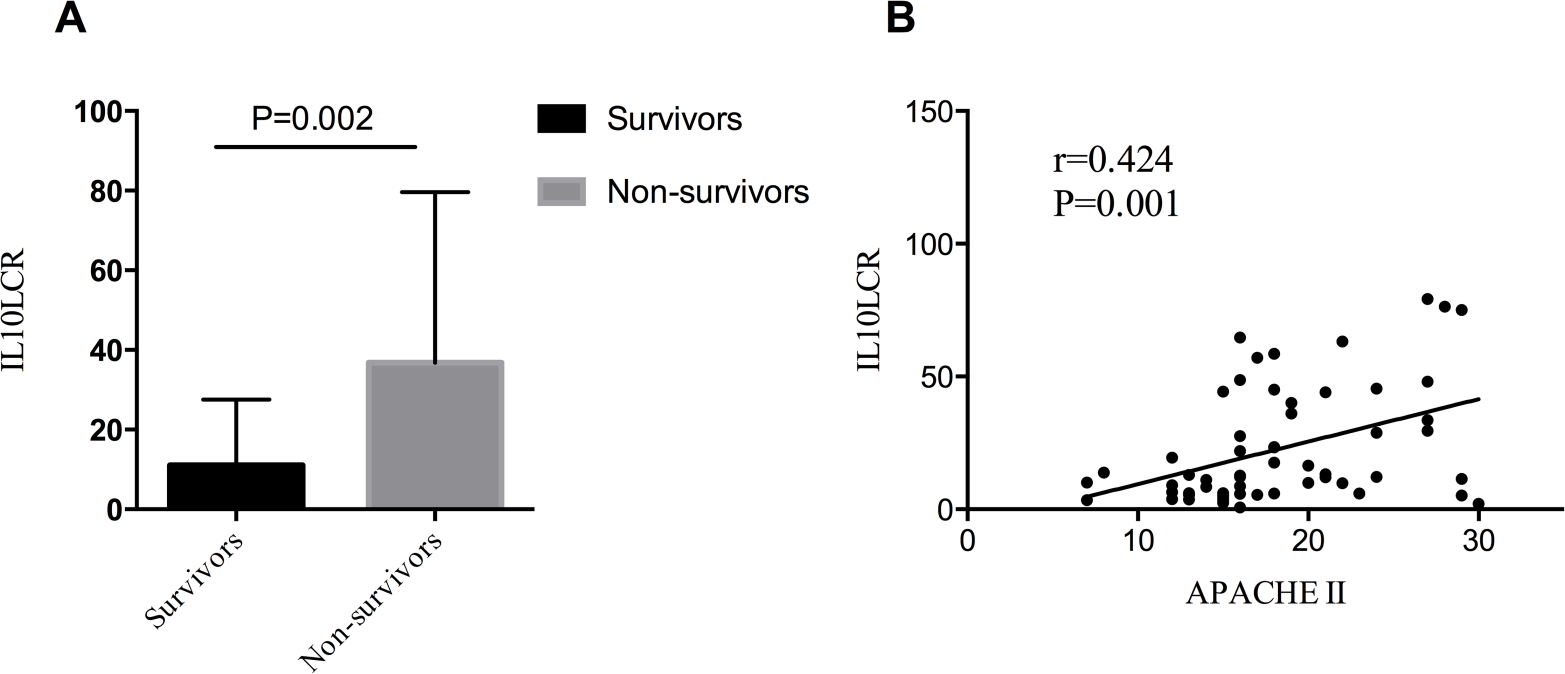
IL10LRC in severity of sepsis. (A) IL10LCR between survivors and non-survivors (median (IQR) 36.78 (12.34–79.63) versus 11.01 (5.41–27.50), *P* = 0.002). (B) IL10LCR was positively correlated with APACHE II score (Spearman’s rho = 0.424, *P* = 0.0009).

As it is shown in Table 1, APACHE II score in non-survivor was higher than the survivor group (median (IQR) 24 (18–31) versus 16 (14–20), P = 0.000). Moreover, IL10LCR was also significantly higher than the survivors (median (IQR) 36.78 (12.34–79.63) versus 11.01 (5.41–27.50), *P* = 0.002)(Fig 2A**)**.

**Table 1:** Comparison of baseline characteristics and clinical data between survivors and non-survivors with severe sepsis.

	Survivors	Non-survivors	*P* value
	n = 43	n = 20	
Age (years), mean±SD	62±16	57±13	0.268
Male, n(%)	24(56%)	12(60%)	0.791
APACHE II	16(14–20)	24(18–31)	0.000
Source of infection, n(%)			
Lung	38(88%)	20(100%)	
Abdomen	2(5%)	0	
Urinary tract	2(5%)	0	
Blood	1(2%)	0	
Underlying diseases or conditions, n(%)			
Hypertension	22(51%)	5(25%)	
Diabetes mellitus	7(16%)	4(2%)	
Chronic renal failure	4(9%)	1(5%)	
Connective tissue disease	1(2%)	1(5%)	
Cerebrovascular attack	2(5%)	1(5%)	
Malignancy	4(9%)	1(5%)	
Temperature (°C), media(IQR)	37.9±1.2	38.0±1.0	0.846
Hate rate (bmp), media(IQR)	123±26	107±33	0.050
Respiration (bmp), media(IQR)	30(26–36)	27(34–43)	0.201
White blood count (×10^9^/L), media(IQR)	12.42(9.00–15.91)	19.30(12.39–28.23)	0.015
Lymphocyte (×10^9^/L), media(IQR)	0.9(0.5–1.3)	0.6(0.4–0.8)	0.051
IL-10 (pg/ml), media(IQR)	9.02(6.73–13.38)	21.07(8.40–53.50)	0.006
IL10LCR (ng/ml^2^), media(IQR)	11.01(5.41–27.50)	36.78(12.34–79.63)	0.002

SD: standard deviation; APACHE II: Acute Physiology and Chronic Health Evaluation; IQR: 25%-75% interquartile range; bmp: beats per minute; *P* value less than 0.05 was considered statistically significant.

### Correlations between levels of IL10LRC and APACHE II

Spearman correlation coefficient was performed to test the correlations between IL10LCR levels and APACHE II. We use the ROUT method for identifying outliers by Prism 5.0. It is showed that the levels of plasma IL10LCR were positively correlated with APACHE II score (Spearman correlation 0.424, *P* = 0.001)(Fig 2B**).**

### Levels of IL10LRC and severe sepsis outcome

**Fig 3:**
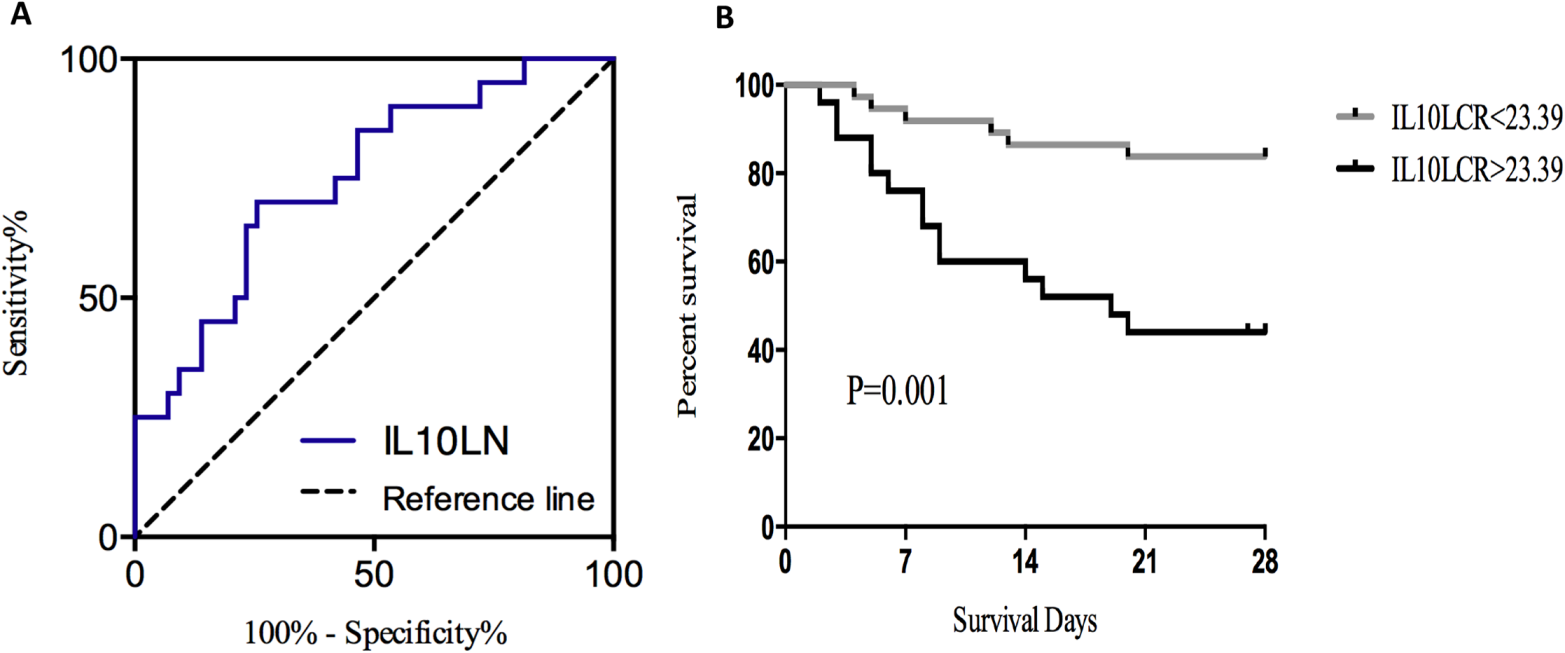
Levels of IL10LRC and severe sepsis outcome. (A) The receiver operating characteristic (ROC) curves showed the area under the curve was 0.749 (95%CI 62.1%-87.7%, *P* = 0.002) for IL10LRC level to predict 28-day mortality with sensitivity and specificity at70.0% and 74.4%, respectively. (B) Patients stratified according to ROC-determined cutoff point of 23.39ng/ml^2^, Kaplan-Meier curve showed survival in patients with IL10LCR level above 23.39ng/ml^2^ was significantly lower than in patients less than 23.39ng/ml^2^ (*P* = 0.001 by log-rank test).

We found that the area under the ROC curve of 0.749 (95%CI 62.1%-87.7%, *P* = 0.002) (Fig 3A). At an optimal cutoff value of 23.39 ng/ml^2^, the sensitivity and specificity of IL10LCR for predicting 28-day mortality were 70.0% and 74.4%, respectively.

Patients were categorized into strata according to IL10LCR level above and below 23.39 ng/ml^2^ as described. Notably, the Kaplan-Meier survival curve showed that the patients with IL10LCR above 23.39 ng/ml^2^ were at greater risks of death than others (*P* = 0.001 by log-rank test) (Fig 3B). This indicated that higher levels of IL10LCR were significantly associated with higher mortality of server septic patients.

For further risk assessment, we performed simple and múltiple Cox regression to analyze hazard ratios (Table 2). Among all collected parameters, APACHE II score, IL10LCR, IL-10 were associated with mortality in simple Cox regression. The hazard ratio and 95% confidence interval (95%CI) for APACHE II score, IL10LCR, IL-10 were 1.211(1.060–1.187), 1.005(1.002–1.008), 1.014(1.007–1.021), respectively. However, only APACHE II score remained significant after adjusting for age and gender in a multiple Cox regression, with a hazard ratio of 1.101 (95%CI, 1.026–1.181).

**Table 2:** Cox proportional hazards models for mortality prediction.

Variable	Simple Cox model		Multiple Cox model	
	HR(95%CI)	*P* value	HR(95%CI)	*P* value
Age	0.984(0.958–1.011)	0.253	NA	NA
Sex	1.129(0.461–2.763)	0.791	NA	NA
IL10LNC	1.005(1.002–1.008)	0.000	0.997(0.988–1.006)	0.500
IL-10	1.014(1.007–1.021)	0.000	1.012(0.991–1.034)	0.269
Lymphocyte	0.381(0.132–1.100)	0.075	NA	NA
APACHE II	1.211(1.060–1.187)	0.000	1.101(1.026–1.181)	0.008

HR: hazard ratio; NA: not application; *P* value less than 0.05 was considered statistically significant.

## Discussion

In the present study, we found that the ratio of IL-10 to lymphocyte in non-survivors,at ICU admission was significant higher than the survivors in severe sepsis. IL10LCR may become a good indicator to predict mortality in severe septic patients.

Sepsis is complex immune response and is accompanied with considerable derangements of both the innate and adaptive immune systems[14]. Invasive infection triggers a pro-inflammatory and anti-inflammatory response, the magnitude of which depends on multiple factors, including pathogen site of infection, virulence, host genetics, and comorbidities[15]. Finally, most septic patients died of immunoparalysis. Recently, some post-mortem studies have shown that the patients who died of sepsis were in the stage of immunoparalysis, mainly displayed in massively depleted immune effector cells and opportunistic infections[16,17]. Therefore, restoring host immunity is a potential way to cure sepsis patients[18]. Hence, it is of great importance to find a good indicator to detect immune status and serve as a predictor of death. The increase of anti-inflammatory cytokines (eg. IL-10) and apoptosis of lymphocyte were the main features during immunosuppression in sepsis[11,19]. Therefore, we combined with IL-10 and lymphocyte to reflect the status of sepsis-induced immunosuppression. Fortunately, we found that IL10LCR was significantly higher than the survivors of severe sepsis (median (IQR) 36.78 (12.34–79.63) versus 11.01 (5.41–27.50), *P* = 0.002). Furthermore, IL10LCR was positively correlated with APACHE II and was related to sepsis mortality.

IL-10 is a molecule with immune-regulatory properties. Secretion of IL-10 in sepsis could limit and ultimately terminate inflammatory responses[20], which called as anti-inflammation cytokine. Besides, high levels of IL-10 could induce a state of functional immunoparalysis, leading to an incontrollable infection[21]. Previous studies demonstrated that overproduction of IL-10 was a predictor of severity and fatal outcome[7,8]. Our study also showed that IL-10 in non-survivors were higher than survivors(Table 1). We also obtained the area under the ROC curve of IL-10 was 0.715 (Date not shown in article), but when combining with lymphocyte, the ROC curve for predicting death increased to 0.749 (95%CI 62.1%-87.7%, *P* = 0.002).

We found lymphocytes in non-survivors were less than in survivors (*P* = 0.051). Previous studies have demonstrated that the level of lymphocyte, mainly B and T cells, decreased in septic non-survivors compared to survivors[10,11,22–25]. Furthermore, Wyllie *et*.*al* highlighted that lymphocytopenia was a useful diagnostic and prognostic marker of bacteremia in adult medical emergency admissions. Lymphocyte count was significantly associated with bacteremia in multivariate analysis[26]. Moreover, previous studies showed that sepsis patients with persistent lymphopenia had a greatly increased risk of nosocomial infections compared to patients whose lymphocyte level recovered to normal[11,27]. In the sepsis model of mice, Hotchkiss and his collogues showed that the prevention of lymphocyte apoptosis resulted in a marked improvement in survival and a therapy of caspase inhibitors which could prevent lymphocyte from apoptosis and improving survival[28,29].

The level of IL-10 and lymphocyte had a close interaction. On one hand, the lymphocytopenia in sepsis resulted from both depletion and apoptosis[30], IL-10 may play a role in the apoptosis of T-lymphocytes[31–33]. On the other hand, T cell and B cell could express and secret IL-10[34]. Therefore, to better understanding the change of physiopathology in sepsis, it is good way to combining with IL-10 and lymphocyte.

### Limitation

Our sample amount is relatively small and is a single center study. It may influence the analysis of multivariate Cox regression model. Besides, as a retrospective study, blood sample was limited to examine other known biomarkers of sepsis-induced immunosuppression, such as monocyte HLA-DR expression, PD-1 on T cells, TNF levels. For this reason, we couldn’t definitely evaluate their relationship with L10LCR and the status of immunosuppression. In the end, a large prospective study should be going on to evaluate whether IL10LCR can serve as a biomarker for sepsis-induced immunosuppression.

## Conclusions

The current study demonstrated that IL10LCR was significantly correlated with APACHE II score and associated with worse outcomes in severe septic patients. It is an indicator to reflect the severity of critical illness and may serve as a biomarker for sepsis-induced immunosuppression.

## Supporting information

S1 Table (XLSX)

S1 File (SAV)

## Abbreviations

ICU: intensive care units
IL-10: Interleukin-10
IL10LCR: Interleukin-10 –lymphocyte count ratio
APACHE II: Acute Physiology and Chronic Health Evaluation II
